# Ramp position for intubating morbidly obese parturient: What’s new?

**DOI:** 10.4103/0019-5049.79881

**Published:** 2011

**Authors:** Chitra Rajeswari Thangaswamy, Lenin Babu Elakkumanan

**Affiliations:** Department of Anaesthesiology and Critical Care, Jawaharlal Institute of Postgraduate Medical Education and Research, Puducherry, India

Sir,

We read with interest the review article regarding difficult airway and positioning of obese parturient for intubation.[1] We would like to add a comment with reference to position, which would benefit the readers. Ramp position is widely accepted for intubating obese patients. Obtaining optimal ramp position using blankets folded under the chest and head would be tedious as it is a trial-and-error method involving adding or removing blankets.[2] Also, it would be troublesome to remove those blankets after the patient has been anaesthetized as it may cause inadvertent disconnection of monitors and breathing circuits, as well as injury to the patient or the operating room personnel. Also, the “head up” position may cause haemodynamic instability after induction of anaesthesia, which might be undesirable in the setting of obstetric emergency. To avoid this, supine position should be achieved as soon as possible after endotracheal intubation, which might take a few moments. To overcome these problems, alternative methods have been described in the literature to achieve ramp position, which include Rapid Airway Management Positioner [RAMP] system and 25-degree “back up” position [TABLE RAMP].[3–4] In both these techniques, one can position and reposition the patient comfortably in less time.

Rapid Airway Management Positioner (RAMP) system is a new positioning system, which has been evaluated in morbidly obese patients for bariatric surgery and found to be effective.[3] This contains specially designed inflatable chambers, which would be filled with compressed air or nitrogen. Because of the need for compressed air or nitrogen, it may not be available in all hospitals. Another alternative would be TABLE RAMP, which is made using the electronic table; it controls and flexes the table at trunk-thigh hinge and raises the trunk portion of the table to optimal position.[4] The head piece of the table may or may not be removed depending upon the patient’s height. This technique has been compared with classical positioning with blankets, and it has been found that both are equivalent. But we believe that the TABLE RAMP has the advantage of making the patient assume supine position immediately; unlike with the blankets, which takes longer time. Operating room table with electronic control would be available in majority of the hospitals. So whenever feasible, TABLE RAMP should be used to achieve ramp position for intubating an obese parturient.
